# Deep-Sea Ecosystems as an Unexpected Source of Antibiotic Resistance Genes

**DOI:** 10.3390/md23010017

**Published:** 2024-12-31

**Authors:** Wei Zhang, Yingdong Li, Yunmeng Chu, Hao Liu, Hongmei Jing, Qianfeng Xia

**Affiliations:** 1 NHC Key Laboratory of Tropical Disease Control, School of Tropical Medicine, Hainan Medical University, Haikou 571199, China; 2200208011@hainmc.edu.cn; 2 Institute of Deep-Sea Science and Engineering, Chinese Academy of Sciences, Sanya 572000, China; ylifc@connect.ust.hk (Y.L.); chuym@idsse.ac.cn (Y.C.); liuh@idsse.ac.cn (H.L.); 3 HKUST-CAS Sanya Joint Laboratory of Marine Science Research, Chinese Academy of Sciences, Sanya 572000, China

**Keywords:** antibiotic resistance genes, short-read sequencing, assembled contig sequencing, evolutionary strategies, deep-sea ecosystems

## Abstract

The deep-sea ecosystem, a less-contaminated reservoir of antibiotic resistance genes (ARGs), has evolved antibiotic resistance for microbes to survive and utilize scarce resources. Research on the diversity and distribution of these genes in deep-sea environments is limited. Our metagenomics study employed short-read-based (SRB) and assembled-contig-based (ACB) methods to identify ARGs in deep-sea waters and sediments and assess their potential pathogenicity. SRB prediction was found to be more effective for studying the abundance and diversity of these genes, while combining both methods better illustrated the relationship of ARGs with the hosts. Deep-sea waters (DSW) and trenches had the highest diversity of ARGs, including β-lactams, multidrug resistance genes, and rifamycins. Mobile genetic elements, such as IncQ and RP4 plasmids, were also identified. The ratio of nonsynonymous to synonymous substitutions (pN/pS) values of these genes suggest different evolutionary strategies in response to deep-sea conditions and possible human impacts. These resistome profiles provide valuable insights into their natural origins as well as the ecological and evolutionary implications of antibiotic resistance in deep-sea ecosystems. The exploration of the global distribution of ARGs in diverse deep-sea environments is a novel approach that will assist in understanding their potential reservoirs and evolutionary mechanisms. Therefore, employing a comprehensive approach to studying ARGs is particularly necessary. Unique microbial life in deep-sea ecosystems, especially in deep-sea cold seeps sediments (DSCSS), deep-sea waters (DSW), and trench waters (TW), could be a valuable source of new antibiotics and resistance discovery.

## 1. Introduction

Microorganisms utilize antibiotics as weapons to eliminate or inhibit competitors, thereby gaining access to limited resources within ecosystems [1,2]. In order to counteract the effects of antibiotics, microorganisms have developed antibiotic resistance mechanisms mediated by antibiotic resistance genes (ARGs), which have co-evolved with microorganisms to neutralize or resist antibiotic effects [3]. In response to the overuse of antibiotics over the past few decades, the prevalence of ARGs in microbial genomes has increased significantly, giving rise to concerns about their potential threat to public health and environmental safety [4]. This raises the critical question of which proto-resistance and resistance elements may emerge to confer resistance to current and future antibiotics.

The use of antibiotics has led to the rapid development of incoming microbial resistance genes. Even in ecosystems with less antibiotic contamination, the arms race between the development of ARGs and antibiotics has evolved over tens of thousands of years [5]. ARGs in diverse ecosystems serve as a natural reservoir of resistance mechanisms against both existing and emerging antibiotics [6]. Consequently, early detection of ARGs from ecosystems with less antibiotic contamination could assist in the prediction of the emergence of proto-resistance and resistance elements in future clinical settings.

Deep-sea ecosystems harbor a diverse array of microbial species with a long evolutionary history and intense competition for resources, potentially nurturing a rich microbial reservoir carrying resistance genes or antibiotics. Previous studies have reported that prokaryotes [7], fungi [8], and viruses [9] carry ARGs in different environments, such as soil, water, and air [10]. Wastewater treatment plants, landfill leachate, agricultural, animal, and industrial sources; and estuaries are the main sources of antibiotics and antibiotic-resistant bacteria and genes [11]. In recent years, a large number of ARGs have also been found in cold seep sediments [12], deep-sea sediments [13], and waters of the South China Sea (SCS) [14]. Investigating the ecology of ARGs in deep-sea ecosystems could enhance our understanding of potential pathogen resistomes and inform the development of more effective therapeutic strategies [15]. In addition, current approaches for environmental ARGs have focused on metagenomic techniques, and methods based on short reads and assembled contigs have been applied to investigate ARGs due to their respective and complementary advantages. Short-read-based (SRB) methods provide high throughput and sensitivity to rapidly assess the diversity and abundance of ARGs but lack comprehensive sequence information [16], while assembled-contig-based (ACB) methods provide comprehensive sequence information and host context of ARGs, which could help identify the association of genes with mobile genetic elements, the potential propagation, and evolutionary strategies as well as discover new genes [17]. To our knowledge, there has been no evolutionary study of deep-sea-derived ARGs. This study aims to reveal the dynamic adaptive ability of ARGs in different environments and the effect of environmental stress on the propagation of ARGs. It aims to expand the cross-field of ecology and evolution of ARGs and provide a new theoretical basis for the prevention and control of antibiotic resistance. Combining short-read and assembled-contig-based approaches can not only enable comprehensive analysis of the distribution and abundance of ARGs but also help explore their host associations and ecological significance and advance understanding of the natural origins and transmission mechanisms of ARGs. However, a comprehensive investigation of ARGs in deep-sea ecosystems on a global scale remains insufficient. By analyzing 1299 metagenomic datasets, we elucidated the distribution patterns, types, hosts, and evolutionary ecology of ARGs in deep-sea ecosystems, including deep-sea cold seeps sediments (DSCSS), deep-sea sediments (DSS), deep-sea waters (DSW), trench sediments (TS), and trench waters (TW). The results obtained from the two methods, ACB and SRB, were compared to identify the most optimal approach for the characterization of ARGs across diverse deep-sea ecosystems.

## 2. Results

### 2.1. Identified ARGs Across Global Sampling Sites

**Figure 1:**
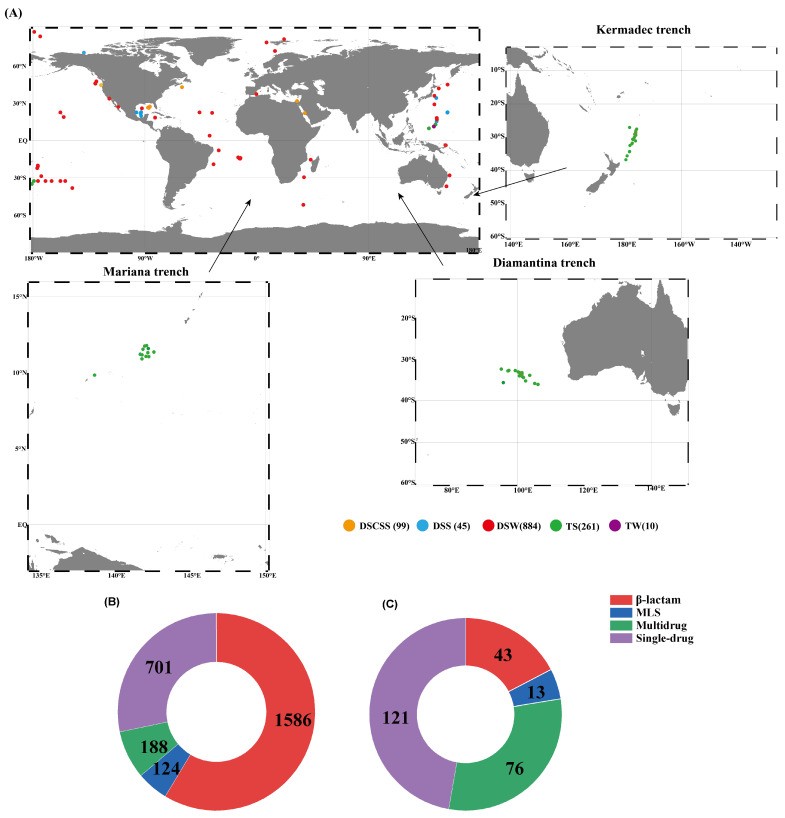
A map of the sample locations. The orange, blue, red, green, and purple circles represent the sites of deep-sea cold seeps sediments (DSCSS), deep-sea sediments (DSS), deep-sea waters (DSW), trench sediments (TS), and trench waters (TW), respectively., and the numbers in parentheses represent the number of samples in the environments (**A**). The distribution of antibiotic resistance genes (ARGs) predicted by the short-read-based (SRB) method (**B**). The distribution of ARGs predicted by the assembled-contig-based (ACB) method (**C**). MSL stands for macrolide–lincosamide–streptogramin.

In total, 1299 metagenomic datasets were compiled from various deep-sea ecosystems globally (Figure 1A). A total of 2599 ARGs and 256 ARGs were identified by the SRB and ACB methods, respectively (Supplementary Data S2). Based on the SRB method, 1586 ARGs (61.1%) were related to β-lactams and the single-drug category (27.0%) (Figure 1B). Conversely, the single-drug category (47.8%) was the major category of ARGs predicted by the ACB method, followed by multidrug resistance ARGs (such as aminocoumarin antibiotics, diaminopyrimidine antibiotics, sulfonamide antibiotics, phenicol antibiotics, penem, and so on, which made up 30.0%) (Figure 1C). 

### 2.2. Distribution Patterns of ARGs Among Deep-Sea Ecosystems

**Figure 2:**
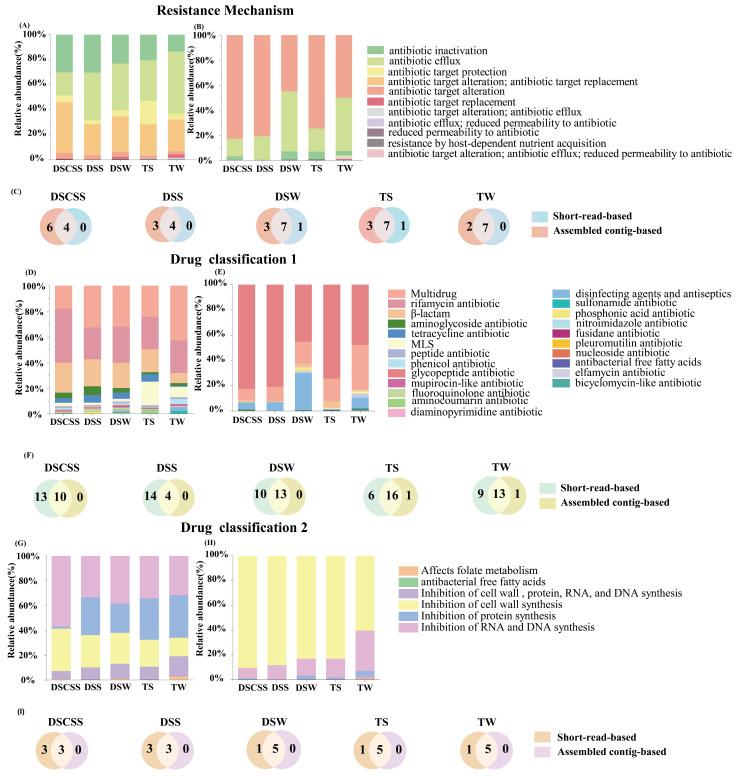
Relative abundance of resistance mechanisms (**A**,**B**), antibiotics (**D**,**E**), and sites of antibiotic inhibition (**G**,**H**) predicted by the SRB and ACB methods in different environments, respectively. Venn plots of ARG types in resistance mechanisms (**C**), antibiotics (**F**), and sites of antibiotic inhibition (**I**) by the SRB and ACB methods in different environments, respectively.

Based on the Comprehensive Antibiotic Resistance Database (CARD) [18], two ARG categories—resistance mechanism (RM) and drug class (DC)—were identified. RMs based on the SRB method contained antibiotic inactivation, antibiotic efflux, and antibiotic target alteration/replacement (Figure 2A). RMs based on the ACB method were predominantly antibiotic target alteration and antibiotic efflux (Figure 2B), with the least number of RM types in DSS (Figure 2C). About seven RM types by the SRB method and four RM types by the ACB method were commonly shared among all ecosystems (Supplementary Figure S1A).

The three most prevalent ARG types identified using the SRB method were rifamycin resistance, β-lactam resistance, and multidrug resistance (Figure 2D). Using the ACB method, the predominant types were glycopeptide resistance and multidrug resistance (Figure 2E). Diversity of DC types based on the SRB method was significantly higher than the ACB method (Welch’s *t*-test, *p* < 0.01) (Figure 2F). About 18 DC types by the SRB method and 4 types by the ACB method were shared among all ecosystems (Supplementary Figure S1B). 

Inhibition of antibiotic DCs could be classified into six types. The major types identified by the SRB method were associated with inhibition of RNA and DNA synthesis, cell wall synthesis, and protein synthesis (Figure 2G), whereas those identified by the ACB method were cell wall synthesis inhibitors and the inhibitors of RNA and DNA synthesis (Figure 2H). Generally, more DC categories were identified by the SRB method than by the ACB method (Figure 2I, Supplementary Figure S1C).

### 2.3. Host Attribution of ARG Classes 

**Figure 3:**
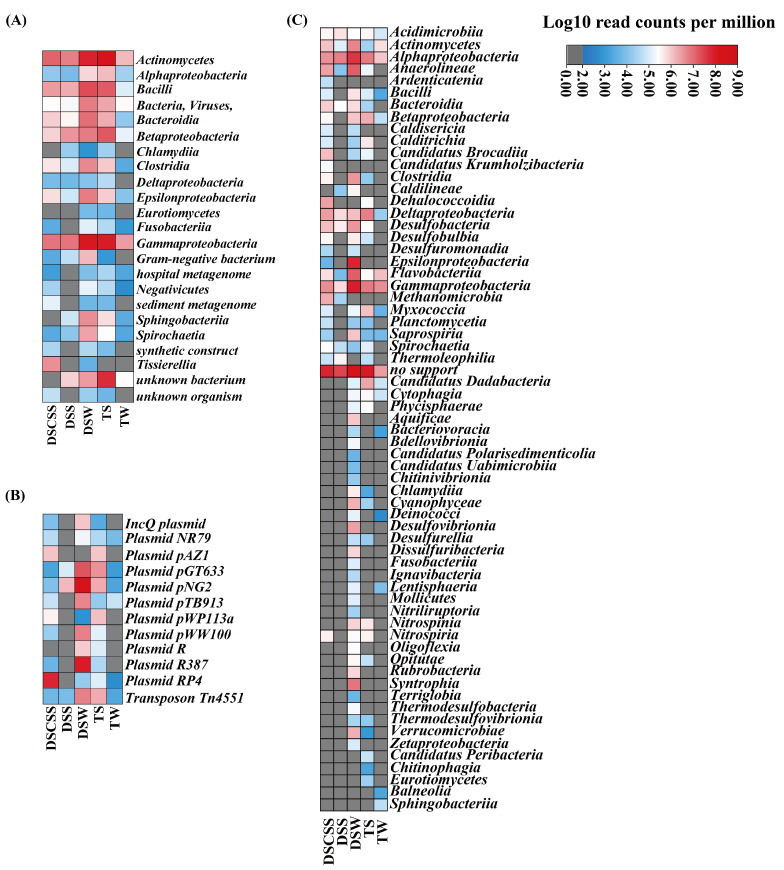
Heatmap showing the relative abundance of hosts and plasmids based on log10-transformed ARG data predicted by the SRB (**A**,**B**) and ACB (**C**) methods in different environments, respectively. Read counts per million is a metric used to normalize high-throughput sequencing data, primarily to convert differences in read counts between samples (e.g., different sequencing depths) into comparable data.

**Figure 4:**
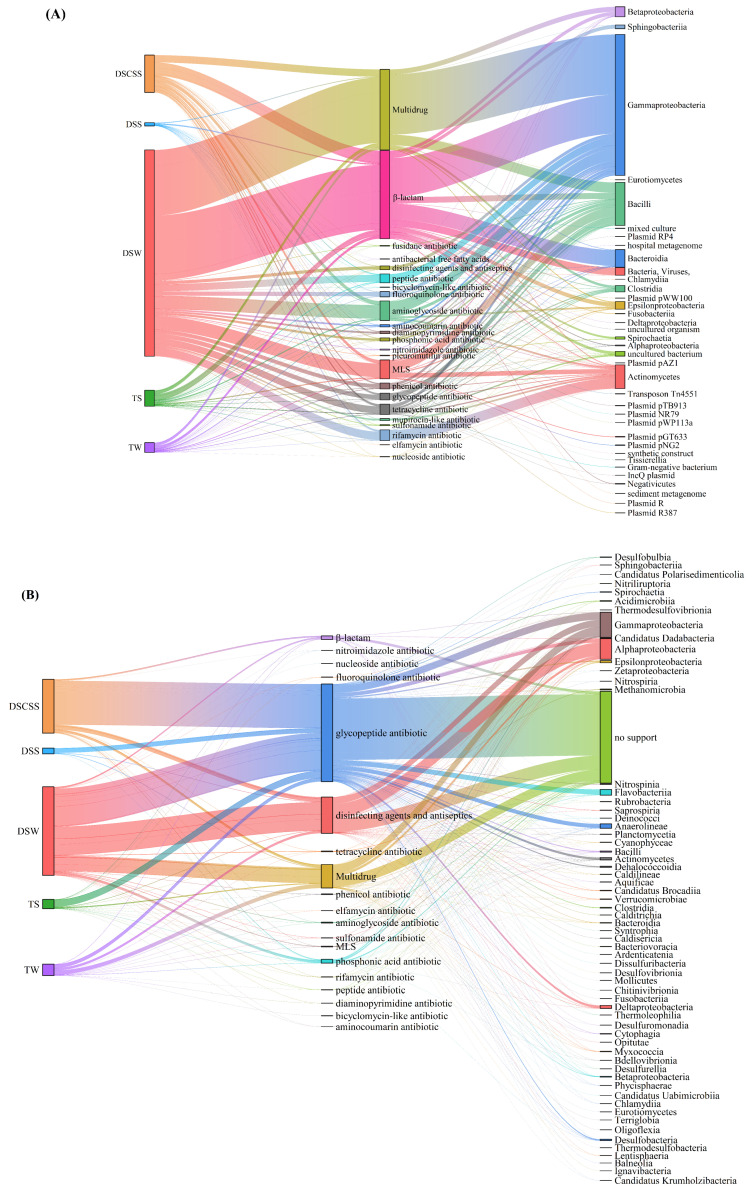
Sankey plots showing the relationship between ARG classes and their hosts by the SRB (**A**) and ACB (**B**) methods in different environments, respectively. The number in parentheses represents the number of host species in the notes in the environment. The wider the width of the flow band, the higher the proportion of ARGs in the corresponding environment or host.

The dominant hosts predicted by the SRB method were *Gammaproteobacteria*, *Actinomycetes*, and unknown bacteria and viruses (Figure 3A). In addition, the plasmids in DSCSS, DSW, and TS showed high diversity. Among them, plasmid pGT633 and plasmid pNG2 were present in these five environments, and InQ plasmid and plasmid RP4 were related to horizontal gene transfer (Figure 3B). Meanwhile, *Gammaproteobacteria*, *Alphaproteobacteria*, *Anaerolineae*, *Deltaproteobacteria*, and unknown bacteria were predicted by the ACB method (Figure 3C). The ACB method identified more hosts in DSCSS than other sites (Figure 4A). The ACB method detected 21 more hosts than the SRB method (Supplementary Figure S2A). Glycopeptide, multidrug, disinfecting agents, and antiseptic ARGs were most prevalent (Figure 4B). Among different sites, DSW exhibited higher host diversity (Supplementary Figure S2B). 

### 2.4. Evolutionary Ecology of ARGs in Deep-Sea Ecosystems

**Figure 5:**
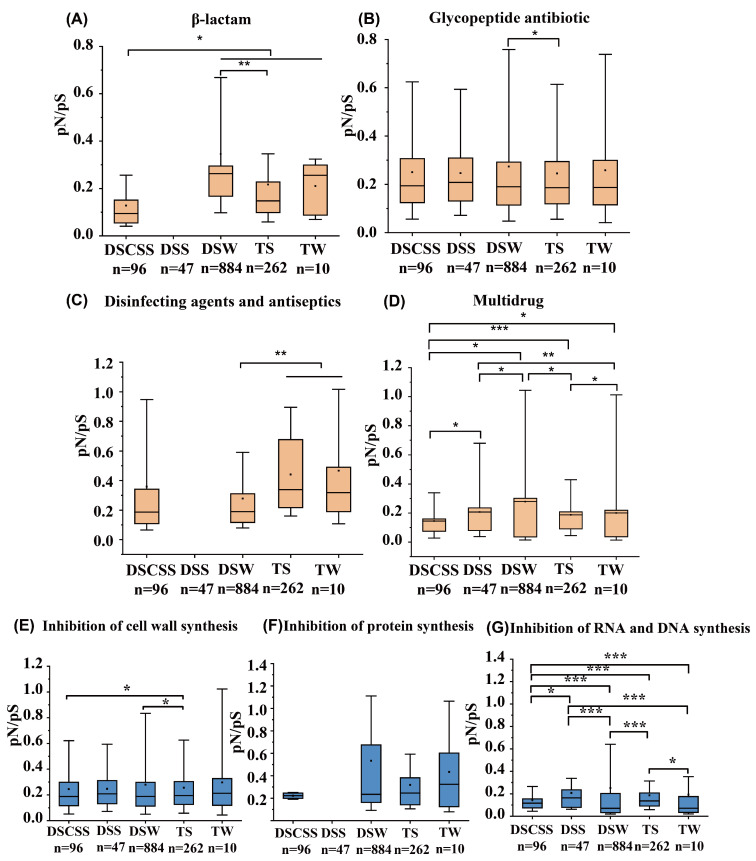
Boxplot of the ratio of nonsynonymous to synonymous substitutions (pN/pS) for ARGs related to β-lactam (**A**), glycopeptide (**B**), disinfecting agents and antiseptics (**C**), multidrug (**D**), inhibition of cell wall synthesis (**E**), inhibition of protein synthesis (**F**), and inhibition of RNA and DNA synthesis (**G**) in different environments. * *p* < 0.05, ** *p* < 0.01, *** *p* < 0.001.

ARGs exhibited considerable variability in the ratio of nonsynonymous to synonymous substitutions (pN/pS) across habitats. Significantly different pN/pS was found between DSW and DSCSS/TS as well as between DSCSS and TS/TW for β-lactam-affiliated ARGs (*p* < 0.05, *Wilcoxon rank sum test*; Figure 5A); between DSW and TS for glycopeptide-affiliated ARGs (*p* < 0.05; Figure 5B); between DSW and TS/TW for disinfecting agents and antiseptic-affiliated ARGs (*p* < 0.05; Figure 5C); between DSCSS and other environments for inhibition of RNA and DNA synthesis (*p* < 0.05; Figure 5G); and between TS and DSW/DSCSS for inhibition of cell wall synthesis (*p* < 0.05; Figure 5E). For multidrug-affiliated ARGs, the pN/pS in DSCSS was significantly lower than in other sites (*p* < 0.05), especially between DSW and DSS/TS as well as between TS and DSW/TW (*p* < 0.05; Figure 5D).

## 3. Discussion

### 3.1. Appropriate Method for ARG Identification

Comparatively higher diversity of RMs and DCs was predicted by the SRB method, which could be used to directly map the CARD database to obtain ARGs and their abundance. This method is well adapted to the increasing number of input (query) sequences and reference data and is able to recognize ARGs from low-abundance organisms present in complex communities [19]. In terms of host prediction, the ACB method would miss these genes due to incomplete or poor assembly and would not annotate the plasmids directly. However, the spliced metagenomic data means the ACB method is able to reconstruct the complete plasmid sequences more efficiently and represent the plasmid types more accurately [19]. The method can also reveal a large number of unknown bacteria, viruses, and rare bacteria that have only recently been discovered [20], making it more appropriate for in-depth analyses of the evolutionary strategy of ARGs, host associations, and the complex structures of resistance genes. Combining these two methods could offer a comprehensive understanding of the distribution and diversity of ARGs and their hosts in deep-sea environments [21]. 

### 3.2. Resistance Mechanisms and Hosts of ARGs 

ARGs are critical for the survival as well as adaptive and resistant evolution of microbes and are essential for managing global antibiotic resistance. In this study, the SRB method could predict more mechanisms, consistent with the finding in other deep-sea environments [14]. Rifamycin resistance, β-lactam resistance, and multidrug resistance were the main ARGs predicted by the SRB method, while glycopeptide resistance and multidrug resistance were the main ARGs predicted by the ACB method. High abundance of ARGs from actinobacteria was detected in DSCSS, where a higher proportion of actinobacteria likely produced rifampicin and rifampicin-related ARGs [22]. Xu et al. evaluated 50 *Actinomycetes* strains derived from the deep sea for their antimicrobial activities against a panel of pathogens, and the results suggest that deep-sea marine *Actinomycetes* represent a promising source of new antimicrobial natural marine products [23]. An increasing number of glycopeptide antibiotic (GPA) producer genomes are being unraveled that carry a large number of differently arranged GPA resistance (named *van*) genes [24,25]. In producing *Actinomycetes*, *van* genes are often associated with antibiotic biosynthesis gene clusters used for GPA biosynthesis and are likely to be transferred/aligned to favor possible co-regulation between antibiotic production and self-resistance [24]. Antibiotic production by *Actinomycetes* also induces the output of corresponding ARGs. For example, the *Actinomycete* genus *Streptomyces spp*. contains multiple resistance genes to avoid damage to itself from spontaneously synthesized antibiotics [26]. ARGs of macrolide–lincosamide–streptogramin (MLS), which are frequently found in human pathogens and are mainly mediated by plasmids and transposons [27], are predominantly in TS and TW. Zhang et al. reported that MLS accounts for a significant proportion (fourth highest) of ARGs in the different deep waters of the Western Pacific Ocean [14]. Trenches are reservoirs for heavy metals [28], microplastics [29], organic pollutants [30,31,32], and ARGs, possibly induced by anthropogenic impacts. The unique funnel-shaped topography, together with the sedimentation effect, may exacerbate the accumulation of contaminants in trenches [33]. The intra-currents may facilitate the circulation of anthropogenic pollutants within the trench [34]. This suggests that remote marine ecosystems could serve as potential reservoirs for resistance genes with natural and anthropogenic influences. Previous studies have also shown that multidrug resistance genes make up a significant proportion of ARGs in deep-sea and hadal environments [35,36]. Even in deep-sea basins of the Western Pacific Ocean, the proportion of multidrug and β-lactam resistance genes could be as high as 49%-100% [36]. Our results indicate that these resistance genes are dominant in various deep-sea environments on a global scale.

Host identification for ARGs is critical to understanding how these genes evolve and spread in the ecosystems as well as tracing the origins of ARGs [37]. *Gammaproteobacteria* and *Actinomycetes* are the two most important potential hosts found in all habitats. *Gammaproteobacteria* is frequently found in many ARG-rich environments, such as hospital wastewater [38], municipal wastewater [39], and fertile soils [40]. *Actinomycetes* are widespread in marine sediments [41], deep-sea cold seeps, and hydrothermal vents [42] and are capable of counteracting the antibiotics they produced [43], with gene sequences similar to those found in clinically pathogenic bacteria [1]. ARGs enable microbes to survive with exposure to antibiotics, while microbes carrying ARGs on mobile genetic elements spread resistance through horizontal gene transfer [44], such as the IncQ plasmid found in DSCSS and DSW, which is characterized by high mobility and is capable of functioning in a variety of bacterial hosts [45]. Meanwhile, the plasmid RP4, which is found in DSCSS, DSW, and TW, is mainly transferred in the manner of horizontal gene transfer [46]. In addition, Wang et al. [47] demonstrated that inter-plasmid ARG transfer is a universal mechanism for plasmids to recruit various ARGs, and Li et al. [48] gave insights into the in situ plasmid transfer under environmental stresses. These previous studies shed light on the potential horizontal gene transfer events between plasmids.

### 3.3. Evolutionary Strategy of ARGs

The pN/pS result highlight adaptive trends in ARGs to different environments. DSCSS represents stable environments with reduced selective pressure, resulting in more conservative ARGs undergoing limited adaptive changes [49]. Conversely, more extreme and variable environments, such as DSW, facilitate higher pN/pS and a rapid adaptation to continuously changing conditions [14]. Significant differences in pN/pS existed between nutrient-poor DSW and nutrient-rich TS for the four key ARGs studied, which highlights the importance of environmental conditions acting as distinct selective pressures in shaping unique ARG persistence and microbial diversity [50].

Resistance genes have different evolutionary features. For example, the faster mutation of antibiotics inhibits cell wall synthesis, which would be helpful to the microbial cell wall stability for resisting the stresses of the deep sea [51], while ARGs inhibiting RNA and DNA synthesis have fewer evolved resistance mutations, which might be the result of the complex horizontal transfer of genes and the dispersal limitation of deep-sea environments [52]. This study highlights the selection pressures and the unique conditions of various deep-sea habitats [53], which lead to different outcomes in the evolution, stability, and adaptability of ARGs in deep-sea ecosystems.

## 4. Materials and Methods

### 4.1. Sample and Metagenomic Datasets Collection

Samples were collected from cold seeps in the South China Sea (SCS) and from three global trenches: the Mariana, Diamantina, and Kermadec Trenches. A total of 7 and 10 push core sediments from cold seeps were obtained from the Haima (16°43′ N, 110°28′ E) and Xisha Trough (18°18′ N, 114°08′ E) in a depth of 12 cm below the seafloor (cmbsf) during the cruises of TS07 and HYDZ6-202102, respectively. About 40 samples were collected from the Mariana Trench (114°8’ E, 142°13’ N) during the TS09 cruise, and 133 and 85 sediment samples were collected from the Kermadec Trench (27°2’ W, 175°38’ S) and the Diamantina Trench (33°52’ W, 106°9’ N) during the TS29 cruise, respectively. The collected sediments were immediately frozen and stored at −80 °C upon arrival aboard the research vessel to preserve their integrity for further analysis. Furthermore, deep-sea metagenomic datasets related to cold seeps, deep-sea sediments, and water were also downloaded from the NCBI database. Detailed information about the collected data used in this study is summarized in Supplementary Data S1.

### 4.2. DNA Extraction and Sequencing

In this study, a total of 275 DNA samples from three layers (i.e., 4 cm as one layer) of each push core were extracted using the DNeasy PowerSoil Pro Kit (Qiagen, Germantown, MD, USA) following the manufacturer’s protocol. The quantity of extracted DNA was determined using the Qubit dsDNA assay kit and a Qubit 2.0 fluorometer (Life Technologies, Carlsbad, CA, USA), while its integrity was assessed through 1% agarose gel electrophoresis. The quality of the DNA was further evaluated with a Nanodrop spectrophotometer. The sequencing libraries were prepared using the NEBNext Ultra DNA Library Prep Kit for Illumina (NEB, Ipswich, MA, USA) and sequenced on an Illumina NovaSeq 6000 platform.

### 4.3. Screening ARG with Short-Read-Based and Assembled-Contig-Based Approaches

Clean short reads were generated by removing adapters, barcodes, poly-N sequences, and low-quality reads from the raw 150 bp paired-end reads (nucleotide positions with a quality score below Q30 were also filtered out to ensure high-quality data) using fastqc (v0.12.1) [54] and the Fastx-toolkit software (v0.0.14). The processed clean reads were then analyzed using the Resistance Gene Identifier (RGI) software (v5.1.1) to identify ARGs [18]. The screening was performed under the Homolog detection and Kraken Metagenomics Assembler (KMA) alignment model with a “strict” algorithm based on CARD. Additionally, the microbial taxonomic affiliations from the RGI screening results were extracted for further downstream analysis and visualization.

The processed clean short reads were assembled into contigs using MEGAHIT with default settings (v1.2.9) [55]. Gene prediction was then performed with Prodigal (v2.6.3) [56] under a meta-algorithm to identify open reading frames (ORFs). The predicted ORFs were subsequently screened using the RGI software to identify ARGs, applying both the Prodigal-Under and Prodigal-Anonymous modes with CARD. This comprehensive analysis included assembled contigs, short contigs, small plasmids, low-quality assemblies, and merged metagenomic reads.

### 4.4. Calculation of Distribution Patterns of ARGs

The abundance of assembled contigs was calculated using CoverM (v0.7.0) [57] in Bowtie2 (v2.5.4) [58] by applying the read counts per million algorithm for cross-sample comparisons. The microbial taxonomy of the identified ARGs was determined using the Contig Annotation Tool (CAT, v0.2.0) [59]. The abundance of identified ARGs was further assigned to their corresponding microbial taxa to elucidate the distribution patterns of ARG-affiliated microbial taxa. 

### 4.5. Calculation of the Evolutionary Matrix

The assembly-based model of the RGI was used to extract the predicted sequences of ARGs from each sample. The short reads were then mapped back to the extracted ARG sequences using Bowtie2. Subsequently, the inStrain software (v1.3.1) [60] was employed to calculate pN/pS in ARGs in different deep-sea ecosystems.

## 5. Conclusions

These findings highlight the need for further exploration of ARGs in deep-sea environments and offer a novel perspective for understanding their potential reservoirs and evolutionary mechanisms. The predicted sequences of ARGs by the SRB and ACB methods offer valuable insights into potential reservoirs and mechanisms of antibiotic resistance in natural ecosystems. The SRB method is suited for identifying the types of ARGs and quantifying their abundance in complex communities, whereas the ACB method can carry out the evolutionary analysis of ARGs and their association with hosts by providing comprehensive gene sequence information. This study not only provides new insights into the methodology for studying environmental ARGs but also reveals the distributional features, evolutionary mechanisms, and possible horizontal gene transfer pathways of ARGs in deep-sea environments.

## Data Availability

The raw sequenced data acquired in this study can be accessed at the NCBI under PRJNA612576, PRJDB6686, PRJEB32776, PRJNA859662, PRJEB32934, PRJEB7866, and PRJEB14154.

## Supplementary Materials

The following supporting information can be downloaded at https://www.mdpi.com/article/10.3390/md23010017/s1, Figure S1: Supplementary Figure S1; Figure S2: Supplementary Figure S2; Data S1: Supplementary Data S1; Data S2: Supplementary Data S2.
