# Supplementary material for: Deep-Sea Ecosystems as an Unexpected Source of Antibiotic Resistance Genes
Source: Mar Drugs. 2024 Dec 31;23(1):17. doi: 10.3390/md23010017 (PMC11766751; doi:10.3390/md23010017)
Supplement: Supplementary file 1 [file marinedrugs-23-00017-s001.zip › Supplementary Figure.pdf]

Supplementary Figure

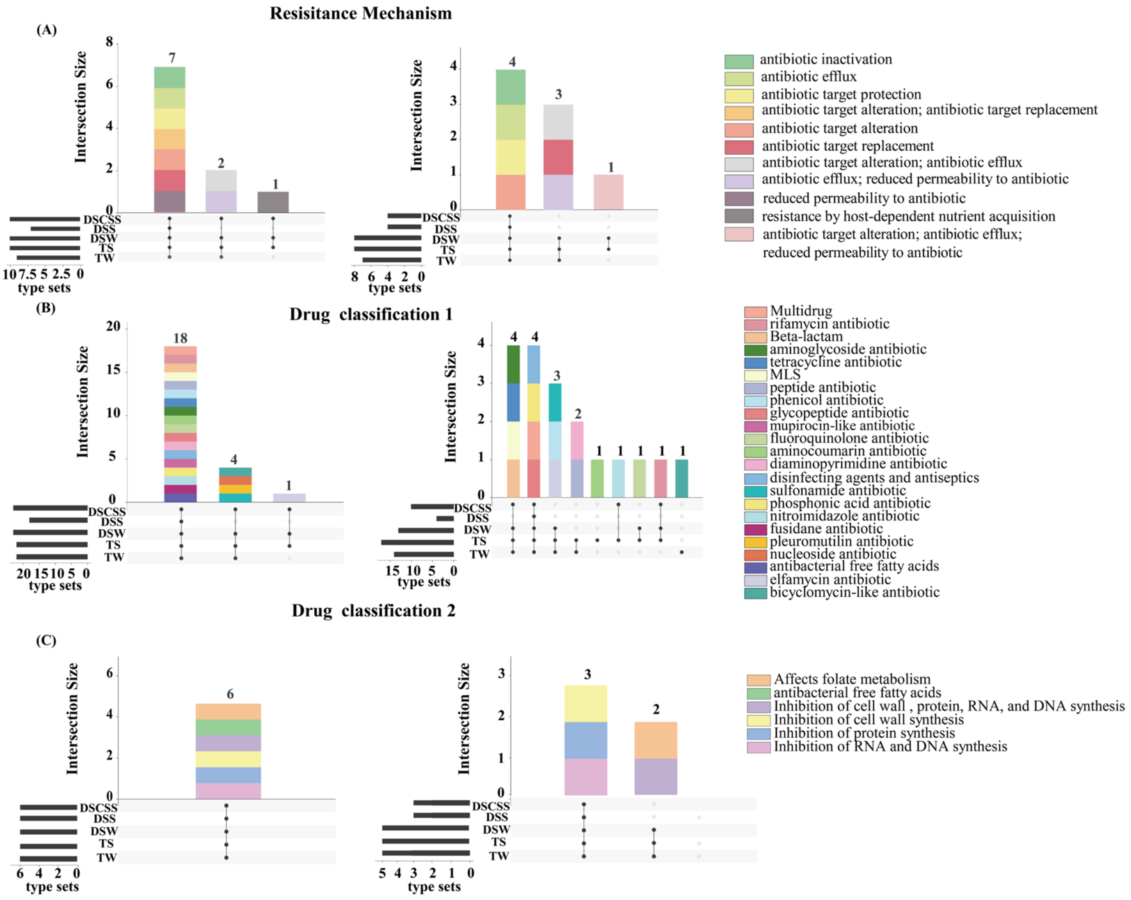

Supplementary Fig.1 Types of resistance mechanisms (A), antibiotics (B), and sites of antibiotic inhibition (C) predicted by the SRB and ACB method in different environments, respectively.

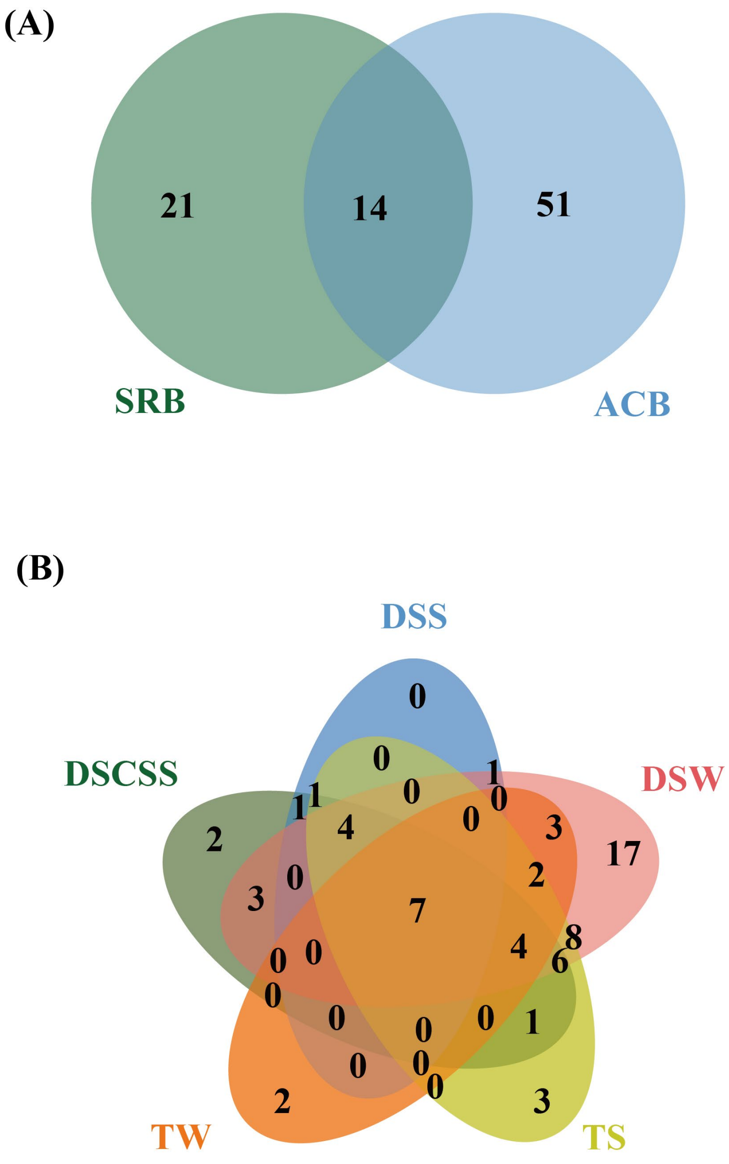

Supplementary Fig.2 Venn plots illustrating host diversity based on the SRB and AB methods (A) and across different environments (B).
